# The Impact of SARS-CoV-2 (COVID-19) on the Acuity of Mental Health–Related Diagnosis at Admission for Young Adults in New York City and Washington, DC: Observational Study

**DOI:** 10.2196/39217

**Published:** 2022-07-14

**Authors:** Amanda Fialk, Alexa Connors, Brianna Cerrito, Karee Jones, Frank Buono

**Affiliations:** 1 The Dorm New York City, NY United States; 2 Department of Psychiatry Yale School of Medicine Yale University New Haven, CT United States

**Keywords:** COVID-19, young adults, mental health treatment, anxiety, depression, youth, mental health, Qualtrics, psychometric properties, gender, mental health service, quality of life

## Abstract

**Background:**

The COVID-19 pandemic has required restrictive measures to mitigate transmission of the virus. Evidence has demonstrated increased generalized anxiety and depression among young adults due to the COVID-19 pandemic. However, minimal research has examined the longitudinal effect of COVID-19 over the course of time and its impact on anxiety and depression. Additionally, age and gender have been found to play a significant role on individuals’ mental health, with young adults and women particularly at risk.

**Objective:**

The aim of this study was to examine the impact of the COVID-19 pandemic on anxiety and depression upon admissions to treatment.

**Methods:**

This was an observational study that was completed longitudinally in which the grouping variable split the time interval into five equal groups for assessments over each period of time. A total of 112 young adults (aged 18-25 years) were recruited for the study. Participants completed assessments online through a Qualtrics link.

**Results:**

Psychometric properties of the admission assessments were uniformly highly statistically significant. There was a significant difference in generalized anxiety between the group-1 and group-3 time intervals. No significant difference was found across the time intervals for depression. Differences in predicting the impact of the psychometrics scores were found with respect to gender. Only the ability to participate and the quality-of-life subfactor of the Functional Assessment of Chronic Illness Therapy (FACIT) assessment were significant.

**Conclusions:**

This study sought to understand the impact that COVID-19 has had on young adults seeking mental health services during the pandemic. Gender emerged as a clear significant factor contributing to increased anxiety in young adults seeking mental health services during the pandemic. These findings have critical importance to ensuring the potential treatment success rate of clients, while providing an overarching understanding of the impact of the pandemic and establishing clinical recommendations for the treatment of individuals who are seeking out treatment.

## Introduction

On March 11, 2020, the World Health Organization declared the coronavirus SARS-CoV-2 (COVID-19) outbreak as a pandemic. To mitigate transmission, restrictive measures were imposed across the United States, including the closure and modification of businesses and mandates for the use of face masks and vaccinations, which have had an immediate and unprecedented impact on psychological health [1-9]. Incidences of depression and anxiety, among other struggles such as substance use, posttraumatic stress disorder (PTSD), eating disorders, and suicidality, have increased as a result of pandemic-related stressors such as lockdowns, exposure to infected people, loss of loved ones, and economic hardship [10,11]. Age and gender have been found to play significant predictive roles on the impact that COVID-19 stressors have had on individuals’ mental health, with young adults and women identified to be particularly at risk [5,6].

Several studies have demonstrated an increased incidence of depression and anxiety during the COVID-19 pandemic among adults [6,12-17]. One US study found that in states that had more than 50 COVID-19 cases since March 10, 2020, each additional day resulted in an 11% increase in the probability of ascending into a higher incidence of mental health crises [18]. Quantitative studies conducted in other countries echo these findings. Ammerman et al [19] investigated the association between COVID-19 and the prevalence of suicidal thoughts and behaviors, showing that 45% of those who endorsed past-month suicidal ideation reported that their thoughts were directly related to COVID-19. Moreover, 9% of the sample reported intentionally exposing themselves to COVID-19, with 50% of these individuals intending to kill themselves through willfully contracting the COVID-19 virus [19]. Taken together, these findings suggest a significant predictive relationship between COVID-19 stressors and the prevalence and severity of individuals’ mental health, including anxiety and depression. However, there is a lack of research examining the impact of COVID-19 over the prolonged course of the pandemic.

Predictive factors related to the impact of COVID-19 stressors have also begun to emerge in the current literature. Age has been found to play a significant predictive role in the impact of COVID-19 on mental health. Evidence from several recent studies suggests that young adults are particularly at risk for experiencing mental health problems during the pandemic [12,14,20-24]. Studies have reported high levels of depression and anxiety symptoms in college students and young adults as a result of COVID-19 pandemic–related stressors [3,5,6,14,16,23,25]. More specifically, researchers evaluating the impacts of the COVID-19 quarantine in France among 69,054 university students found a high prevalence of suicidal ideation (11.4%), severe distress (22.4%), heightened perceived stress (24.7%), severe depression (16.1%), and high levels of anxiety (27.5%) [23]. Researchers also examined the prevalence of PTSD, depression, and psychological risk factors in 2485 home-quarantined college students, finding that PTSD had a prevalence of 2.7% and depression had a prevalence of 9.0% [25]. García-Portilla et al [26] examined the psychological effects of the pandemic across age groups and found that those under 60 years old were at greatest risk. One potential related factor contributing to this finding is lack of resilience, as younger individuals have less years of experience in coping with hardship, which is a developmentally acquired skill [26,27]. These findings suggest that age has the potential to be a strong predictive factor of interest in the relative impact of COVID-19 stressors on key facets of mental health.

Gender has also been found to play a significant predictive role in the relative impact of COVID-19 on mental health. Turna et al [28] examined the effects of the COVID-19 pandemic on multiple facets of mental well-being. Anxiety and depression were assessed using the General Anxiety Disorder (GAD-7), Patient Health Questionnaire (PHQ-9), and Perceived Stress Scale among 632 participants, 82% of whom identified as female. The results showed that nearly one-third (31%) of participants met the criteria for generalized anxiety disorder and 29% met the criteria for major depressive disorder. Female gender was significantly predictive of psychiatric symptoms at a 99% CI. These findings are also supported by evidence found in a study by Emery et al [29] that looked at how COVID-19 impacted the mental health and behaviors of young adults in the United States: female gender, prepandemic mental illness symptoms, and self-reported stress were found to be emergent risk factors for mental illness. Kecojevic et al [30] obtained similar findings when examining the impact of COVID-19 on the mental health of college students, in which female students reported higher anxiety than male students. Collectively, these results suggest that female gender is a risk factor for COVID-19–related anxiety [28-30].

Emerging adulthood is a time of uncertainty and exploration, and the COVID-19 pandemic has had a compounding effect on this process, resulting in a significant decline in life satisfaction and a significant increase in mental health problems [31]. The aim of this study was therefore to examine the longitudinal impact of the COVID-19 pandemic on anxiety and depression upon admissions to treatment at a long-term and intensive outpatient mental health program for young adults (The Dorm). Specifically, this study examined the prevalence and acuity levels of generalized anxiety and depression diagnoses upon admissions in the first 15 months of the COVID-19 epidemic. Particular attention was paid to how gender identity was associated with acuity levels of anxiety and/or depression. We anticipated that as the pandemic continued over time, so would depression and generalized anxiety among young adults.

## Methods

### Participants

We attempted to recruit 133 individuals for this study, 112 of whom ultimately volunteered. The 21 individuals who did not complete the study cited lack of interest for not participating. Among the 112 young adults that agreed to participate (mean age 22.3, SD 3.2 years), 53 (47.3%) self-identified as female. Inclusion criteria were: (1) admitted to The Dorm, (2) provided informed consent, and (3) at least 18 years of age. Exclusion criteria were: (1) unfit to complete the survey due to medical or psychological constraint and (2) not fluent in the English language.

### Setting

The Dorm is an intensive outpatient program for young adults aged 18-35 years operating in New York City and Washington, DC. Empirically supported behavioral psychosocial methodologies are implemented to serve a variety of mental health illnesses and co-occurring disorders over the course of a phased treatment, which is typically a 1-year admittance (on average). In addition, holistic approaches such as exercise, yoga, Reiki, horticulture, community service, meditation, and mindfulness are an integral part of the treatment model. All clients participate in family programming, including weekly parent coaching, parent groups, and family groups. Clients work with both a therapist and a clinical coach, and participate in 3-30 hours a week of group therapy depending on the treatment phase. 

### Assessments

#### Generalized Anxiety Disorder

The GAD-7 is a 7-item, self-rated scale developed in correspondence with the Diagnostic and Statistical Manual and updated for the 5th Edition, which is used as a screening tool and severity indicator for generalized anxiety disorder. Reliability of this assessment demonstrated a Cronbach α of .92 [32].

#### Patient Health Questionnaire

The PHQ-9 is a 9-item self-report scale that is used for screening, diagnosing, monitoring, and measuring the severity of depression (mild, scores of 5-9; moderate, scores of 10-14; moderately severe, scores of 15-19; severe, scores of 20-21) [33]. Reliability of this assessment demonstrated a Cronbach α of .89.

#### Barratt Impulsivity Scale

The Barratt Impulsivity Scale (BIS-11) is a 4-point Likert scale from “rarely/never” (score=1) to “almost always/always” (score=4), where 4 indicates the most impulsive response [34,35]. However, there is no standard BIS-11 score that is consistently used to designate an individual as “highly impulsive.”

#### Functional Assessment of Chronic Illness Therapy Measurement System

The Functional Assessment of Chronic Illness Therapy (FACIT) Measurement System is a 27-item questionnaire that focuses on the domains of physical well-being, social/family well-being, emotional well-being, and functional well-being. Items are scored on a 5-point Likert scale, where participants endorse “not at all” to “very much” as the statement applies over the past 7 days [36].

#### Patient-Reported Outcomes Measurement Information System Scales

##### Ability to Participate in Social Roles and Activities

The Patient-Reported Outcomes Measurement Information System (PROMIS) Ability to Participate in Social Roles and Activities is an 8-item self-report questionnaire that assesses the perceived ability to perform one’s usual social roles and activities. Items are worded negatively in terms of perceived limitations and responses are reverse-coded, where 5 indicates “never” and 1 indicates “always”; thus, higher scores represent fewer limitations (better abilities). The item bank does not use a time frame (eg, over the past 7 days) when assessing the ability to participate in social roles and activities.

##### General Self-Efficacy

PROMIS General Self-Efficacy is a 10-item measure used to assess a person’s belief in their capacity to manage daily stressors and have control over meaningful events [33]. This measure was derived from the National Institutes of Health Toolbox Self-Efficacy Item Bank by creating new “confidence” response options that mirrored the same response options as in the PROMIS measures of the Self-Efficacy for Managing Chronic Conditions scale: “I am not at all confident,” “I am a little confident,” “I am somewhat confident,” “I am quite confident,” “I am very confident” [37].

##### Friendship (Ages 18+) Fixed Form

PROMIS Friendship (Ages 18+) Fixed Form is an 8-item scale evaluating the perceptions of the respondent on their availability of friends or companions with whom to interact or affiliate with in the past month on a 5-point Likert scale (1=never, 5=always).

##### Perceived Rejection (Ages 18+)

PROMIS Perceived Rejection (Ages 18+) is an 8-item scale evaluating how often people perceive others to be arguing/yelling at them and the perceived insensitivity over the past month on a 5-point Likert scale (1=never, 5=always).

#### Sleep-Related Impairment Questionnaire

The PROMIS Sleep-Related Impairment Questionnaire is an 8-item questionnaire that measures self-reported alertness, sleepiness, tiredness, and functional impairments associated with sleep problems during waking hours within the past 7 days [38]. This measure uses a 5-point Likert scale (1=not at all, 5=very much).

### Procedure

All potential participants were consented either through a private meeting using Zoom virtual conference software or in person under standardized COVID-19 protocols. Consent forms were read orally, and any questions were answered by the investigator or research assistant before asking the participant to electronically sign their name on the tablet. By electronically signing their name, the participant was assigned a random 6-digit number generated by Qualtrics survey management tools. All consented individuals were tracked and followed through a password-protected Microsoft Access file. Each participant was asked to complete the assessments online through the Qualtrics link. The research assistant stayed with the participants and provided assistance as needed until the completion of the survey. The total length of time required to complete the survey did not exceed 40 minutes.

### Ethical Considerations

The study was approved by Yale School of Medicine’s institutional review board prior to participant recruitment (IRES number 2000026514). This research was performed in alignment with the World Medical Association Declaration of Helsinki (2018).

### Data Analysis

Data were analyzed using SPSS for Windows, version 26.0. The grouping variable split the time interval into five equal phases (1=March 2020 through May 2020; 2=June 2020 through August 2020; 3=September 2020 through November 2020; 4=December 2020 through February 2021; 5=March 2021 through May 2021).

Mean group and overall differences were analyzed using one-way analysis of variance, whereas dichotomous variables (eg, age, gender, location) were analyzed using Fisher-exact or *χ*^2^ tests. Strengths of association between variables were examined by the Pearson correlation coefficient and by linear regression analyses with generation of odds ratios and accompanying 95% CIs. All statistical tests were two-tailed with an α level of .05. Additionally, linear regression was used with gender (male, female, transgender, and other) as the dependent variable to evaluate its impact on psychometric assessments.

## Results

The demographic characteristics of the participants are summarized in Table 1. Over 98% of participants were single at admission and the majority either had some college or a high school/equivalent education when combined. The majority of participants were white, with equal representation of self-identified males and females at admission, along with individuals identifying as transgender and other represented. The highest percentage of admissions were unemployed 45%, and nearly 60% of those admitted had experienced at least a singular traumatic event in their lifetime.

**Figure 1:**
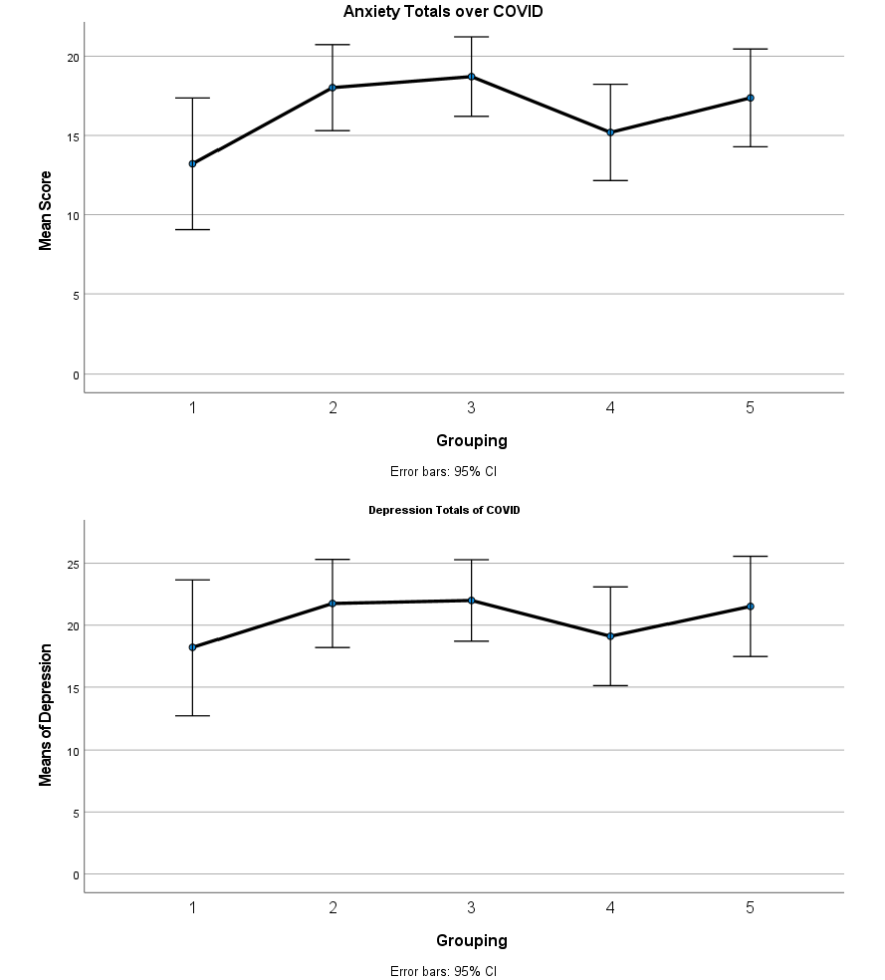
Aggregate differences of anxiety and depression across the past five consecutive quarters.

Table 2 presents a correlation matrix of the psychometric properties of the admission assessments, which were uniformly highly statistically significant. Figure 1 presents the participants’ average acuity of primary diagnosis of generalized anxiety and/or depression across the time intervals, along with the range of scores within each grouping. There was a significant difference in generalized anxiety levels between the group-1 and group-3 time intervals. No significant difference was noted across the time intervals for the depression assessment.

Additionally, the findings from the linear regression demonstrated differences across gender and the admission psychometrics. Significant findings were noted (*F*_101_=2.025, *P*=.04) to predict the impact of the psychometrics scores on gender. However, as noted in the coefficients subtable, only ability to participate (*P*=.002) and the quality-of-life subfactor of the FACIT (*P*=.02) assessments were significant with other values being not significant. In contrast to initial indications, depression and anxiety were not significant factors across gender at admission to this program.

**Table 1:** Demographics of admission intakes (N=112).

Characteristic			Value		*P* value
Age (years), mean (SD)			22.2 (3.2)		N/A^a^
**Gender, n (%)**					.44
	Male	53 (47.3)			
	Female	53 (47.3)			
	Transgender	1 (0.8)			
	Other	5 (4.5)			
**Race, n (%)**					.11
	White	84 (75.0)			
	Black	9 (8.0)			
	Hispanic	5 (4.5)			
	Asian	14 (12.5)			
**Marital status, n (%)**					.10
	Single	110 (98.2)			
	Married	2 (1.8)			
**Highest education, n (%)**					.74
	Some high school	4 (3.6)			
	High school graduation/GED^b^	44 (39.3)			
	Some college	46 (41.1)			
	Associates degree	2 (1.8)			
	Bachelor’s degree	18 (16.1)			
**Employment, n (%)**					.47
	Full time work	14 (12.5)			
	Student	47 (42.0)			
	Unemployed	51 (45.5)			
**Experienced trauma in life, n (%)**					.46
	Yes	67			
	No	45			

^a^N/A: not applicable.

^b^GED: General Educational Development.

**Table 2:** Correlational table of psychometric measures at admission.

Variable			GAD-7^a^ total		PHQ-9^b^		FACIT^c^ physical		FACIT well-being		Ability to participate		Impulsivity		Friendship		Rejection		Self-efficacy		Sleep
**GAD-7 total**																					
	*r*	1		0.740		0.643		–0.421		–0.594		0.077		–0.131		0.381		–0.306		–0.110	
	*P* value	—^d^		<.001		<.001		<.001		<.001		.42		.17		<.001		<.001		.25	
**PHQ-9**																					
	*r*	0.740		1		0.749		–0.579		0.650		0.036		–0.290		0.395		–0.395		–0.080	
	*P* value	<.001		—		<.001		<.001		<.001		.71		.002		<.001		<.001		.40	
**FACIT physical**																					
	*r*	0.643		0.749		1		–0.398		0.575		0.129		–0.084		0.356		–0.251		–0.080	
	*P* value	<.001		<.001		—		<.001		<.001		.17		.38		<.001		.007		.40	
**FACIT well-being**																					
	*r*	–0.421		–0.579		–0.398		1		–0.523		0.525		0.551		–0.196		0.618		0.237	
	*P* value	<.001		<.001		<.001		—		<.001		<.001		<.001		.04		<.001		.01	
**Ability to participate**																					
	*r*	0.594		0.650		0.575		–0.523		1		–0.029		–0.320		0.417		–0.356		–0.109	
	*P* value	<.001		<.001		<.001		<.001		—		.76		<.001		<.001		<.001		.25	
**Impulsivity**																					
	*r*	0.077		0.036		0.129		0.525		–0.029		1		0.345		0.189		0.454		0.149	
	*P* value	.42		.71		.17		<.001		.76		—		<.001		.04		<.001		.11	
**Friendship**																					
	*r*	–0.131		–0.290		–0.084		0.551		–0.320		0.345		1		–0.228		0.544		0.193	
	*P* value	.165		.002		.38		<.001		<.001		<.001		—		.02		<.001		.04	
**Rejection**																					
	*r*	0.381		0.395		0.356		–0.196		0.417		0.189		–0.228		1		–0.121		0.000	
	*P* value	<.001		<.001		<.001		.04		<.001		.04		.02		—		.20		>.99	
**Self-efficacy**																					
	*r*	–0.306		–0.395		–0.251		0.618		–0.356		0.454		0.544		–0.121		1		0.316	
	*P* value	<.001		<.001		.007		<.001		<.001		<.001		<.001		.20		—		<.001	
**Sleep**																					
	*r*	–0.110		–0.080		–0.080		0.237		–0.109		0.149		0.193		0.000		0.316		1	
	*P* value	.25		.40		.40		.01		.25		.11		.04		>.99		<.001		—	

^a^GAD-7: 7-item General Anxiety Disorder scale.

^b^PHQ-9: 9-item Patient Health Questionnaire.

^c^FACIT: Functional Assessment of Chronic Illness Therapy.

^d^Not applicable.

## Discussion

### Principal Findings

Restrictive measures put in place to mitigate transmission of the coronavirus SARS-CoV-2 (COVID-19) have had an immediate and unprecedented impact on people’s psychological health and well-being, especially as they relate to the incidences and severity of depression and anxiety. This study sought to understand the impact that COVID-19 has had on young adults seeking mental health services at an outpatient program during the pandemic. The results indicated a significant increase in anxiety in young adults across the measured time periods of the pandemic, in particular between March and November of 2020, or phases 1-3 as defined in this study. This increase in anxiety coincides with a rising number of cases and COVID-19 becoming the third leading cause of death in the world, as death tolls were hitting their highest peak since 2015 (15.9% increase) [39]. The American Psychiatric Association issued a public opinion poll in 2020, which found that 80% of participants were either somewhat or extremely anxious about keeping themselves and their families safe in 2020, while 75% were worried about COVID-19 specifically.

The results also demonstrated that self-identified gender was a significant predictor of mental health. Female clients scored lower than their male counterparts on measures of “quality of life” and “ability to participate in daily activities,” both of which are subscores that are reliably indicative of broader mental well-being. This is consistent with the literature, which suggests that female-identified individuals are more significantly impacted by psychological distress than their male-identified counterparts [40-42]. In particular, female-identified individuals between the ages of 26-35 years are most at risk in terms of vulnerability to stress [40]. Future research should consider the biological, social, and cognitive mechanisms underlying these gender differences.

### Comparison With Prior Work

Mental health conditions account for 16% of the global burden of disease and injury in people aged 10-19 years. Young adulthood is a critical time of interpersonal, intrapersonal, social, educational, and vocational development [43]. Research shows that the brain does not fully develop until the age of 25, and risk factors for mental health may be of increased concern for the developing brain [8]. Half of all mental health disorders in adulthood start by age 14 [44]. Furthermore, COVID-19 has only exacerbated these statistics, heightening the public health urgency [12,14,20-23]. While young adults were at lower risk for the direct health effects of hospitalization or death due to COVID-19, research is needed to understand any long-lasting mental health effects that may be detrimental to development. The findings of this study echo the need to understand the long-term public health impact on young adult mental well-being. Future research should continue to evaluate the public health impact of COVID-19 on young adults in a systematic manner. 

The findings emphasize the importance of secondary mental health concerns among young adults, especially female clients in this case. While this study demonstrated that young adults experienced a significant increase in anxiety during the pandemic and that gender was a significant factor in the compromised mental well-being of young adults, it will be necessary to continue to study the lasting and secondary impact of anxiety to prepare for and provide successful treatment to young adults. Anxiety is correlated with and can exacerbate secondary mental health concerns and symptoms such as substance use, PTSD, and suicidality. For instance, the prevalence of PTSD in 2485 home-quarantined college students was reported to be 2.7% [25]. In a study that looked at the association between COVID-19 and the prevalence of suicidal thoughts and behaviors, researchers found that 45% of those who demonstrated past-month suicidal ideation reported that their thoughts were directly related to COVID-19 [19]. Finally, substance use may have increased significantly during COVID-19, especially among those who already had preexisting mental health conditions such as anxiety and depression [45,46]. These secondary mental health concerns have the potential to compromise young adults beyond their psychological and emotional well-being. Secondary mental health concerns can lead to reduced productivity at home, school, and in the labor market. Special attention to the secondary effects of heightened anxiety in young adults because of COVID-19–associated stressors is vital to ensure the critical need for mental health preparedness from a global perspective. 

### Limitations

There are several limitations of this study. The longitudinal research design limits the generalizability of the findings due to not having a repeated measurement. Future research should evaluate the long-term impact of similar findings in a more systematic manner that includes a more rigorous research design methodology. Additionally, the sample size is relatively small, thus providing an initial understanding of the acuity of anxiety and depression in young adults seeking intensive outpatient programming during the pandemic. Another limitation is the lack of racial and socioeconomic diversity within the sample. A majority of the sample were white individuals. Additionally, research has demonstrated that marginalized populations such as non-white Hispanic and Asian populations experienced higher levels of the subjective perception of distress, worry, and fear [47]. Further research is needed to explore the impact of COVID-19 on racially and ethnically diverse populations as well as those with different socioeconomic statuses. Additionally, we did not collect information on the socioeconomic status of the participants. Future research is needed to understand if this is a covariate for increased anxiety and depression. While we recognize this as a contributing limitation within the manuscript, it should be noted that longitudinal data discussing the impact of COVID-19 on mental health are currently lacking. Lastly, due to restrictions put in place by COVID-19 and the corresponding state regulations, treatment for individuals within the first quarter was primarily conducted virtually instead of in person. While this deviates from the current protocol in place, all attempts were made to make the virtual sessions as equally representative as the in-person interactions.

### Conclusion

This study attempted to understand the impact of COVID-19 on the admissions of an outpatient program for young adults. It was apparent that COVID-19 had a larger impact on the quality of life for women than for men. Additionally, it was apparent that there was a significant difference in generalized anxiety between the first and third intervals during the COVID-19 pandemic. These findings highlight the need to better understand the mental health impact of the COVID-19 pandemic.

## Abbreviations

BIS-11: Barratt Impulsivity Scale
FACIT: Functional Assessment of Chronic Illness Therapy
GAD-7: Generalized Anxiety Disorder scale
PHQ-9: Patient Health Questionnaire
PROMIS: Patient Reported Outcomes Measurement Information System
PTSD: posttraumatic stress disorder
